# Molecular Dynamics Studies on *Trypanosoma cruzi* Dihydroorotate Dehydrogenase Complexes: An Analysis of the Inhibitor Influence

**DOI:** 10.1021/acsomega.5c01872

**Published:** 2025-04-25

**Authors:** Eldio G. Santos, Luiz A. P. Flores-Junior, Camilo H. S. Lima, Luiza R. S. Dias

**Affiliations:** 1 Laboratório de Química Medicinal, Departamento de Tecnologia Farmacêutica, Faculdade de Farmácia, Universidade Federal Fluminense, Niterói, RJ 24241-000, Brazil; 2 Laboratório de Modelagem Molecular, Departamento de Química Orgânica, Instituto de Química, Universidade Federal do Rio de Janeiro, Rio de Janeiro, RJ 21941-909, Brazil

## Abstract

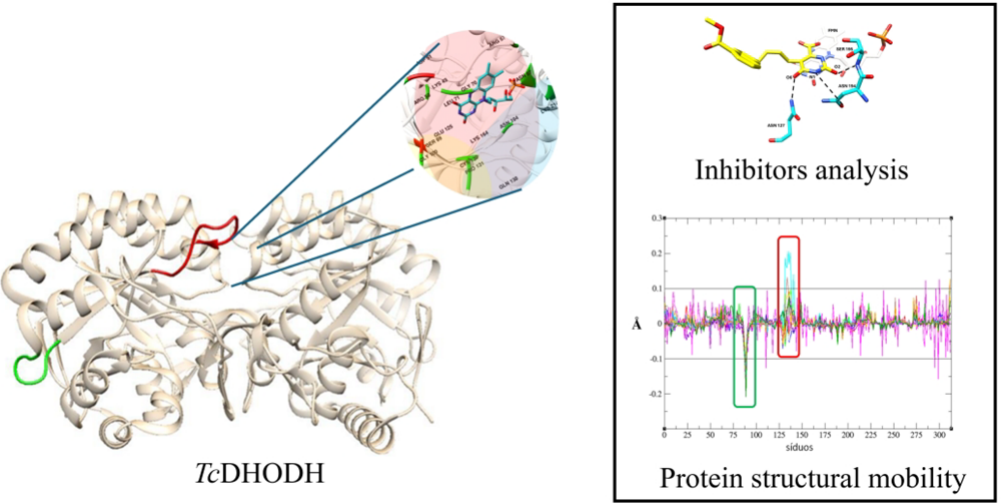

Chagas disease remains a significant global health problem. Current etiological treatment is limited due to its low efficacy in the advanced stage of the disease and adverse effects. *Trypanosoma cruzi* dihydroorotate dehydrogenase (*Tc*DHODH) is a promising target for developing new drugs. This study explored the structural and dynamic factors influencing its inhibition. The results from the 100 ns molecular dynamics simulations of 11 ligand–*Tc*DHODH complexes revealed that ligand size and conformation play crucial roles in enzyme inhibition, with flexibility in the active site being essential for enzyme function. Small ligands tend to maintain a closed conformation, while larger ligands induce open conformations. The results further demonstrate ligand-induced conformational changes and the role of key hydrogen bonds in stabilizing the ligand–enzyme complex. Electrostatic and hydrophobic interactions between ligands and the enzyme’s S1, S2, and S3 subsites contribute to inhibition. Understanding these factors facilitates the development of potent and selective *Tc*DHODH inhibitors for the treatment of Chagas disease.

## Introduction

Chagas disease, a neglected tropical disease caused by *Trypanosoma cruzi*, remains a significant global health concern.^1^ Despite existing treatments, there is an urgent need for new, more effective drugs.^2^ Recent research has focused on exploring novel targets, including enzymes involved in ergosterol biosynthesis^3^ (CYP51), protein degradation^4^ (cruzain), triosephosphate isomerase energy metabolism^5^ (TPI), and nucleotide synthesis^6^ (DHODH). These efforts aim to develop innovative therapies that address the limitations of current options.

Class 1A DHODH is a promising drug target for *T. cruzi*. Its essentiality for parasite survival and structural differences from the human enzyme (*Hs*DHODH) offer potential for selective inhibition.^7^ The *Tc*DHODH enzyme catalyzes a key step in pyrimidine biosynthesis, using flavin mononucleotide (FMN) as a cofactor.^8^ Its homodimeric structure and a unique mechanism involving a catalytic loop make it an attractive target for inhibitor development.^9−11^

Computational methods offer a promising approach to expedite the development of new antichagasic drugs.^12,13^ By targeting key enzymes in *T. cruzi*, these methods can reduce costs and risks associated with traditional drug discovery.^14,15^ To date, studies of *Tc*DHODH described in the literature have mainly focused on structural and functional analyses using techniques such as X-ray crystallography and magnetic resonance spectroscopy. Although the 3D structure of the protein is available, there is a lack of simulation studies on *Tc*DHODH covering molecular dynamics.^15−21^

Given the importance of *Tc*DHODH as a potential Chagas disease target, this study aimed to elucidate the structural and dynamic factors influencing its inhibition by in silico simulations. While previous simulations have explored reaction mechanisms and ligand interactions,^15,22^ a deeper understanding of the *Tc*DHODH-inhibitor complex’s dynamic behavior is needed.

## Results and Discussion

### Crystal Structure Analysis

**Figure 1:**
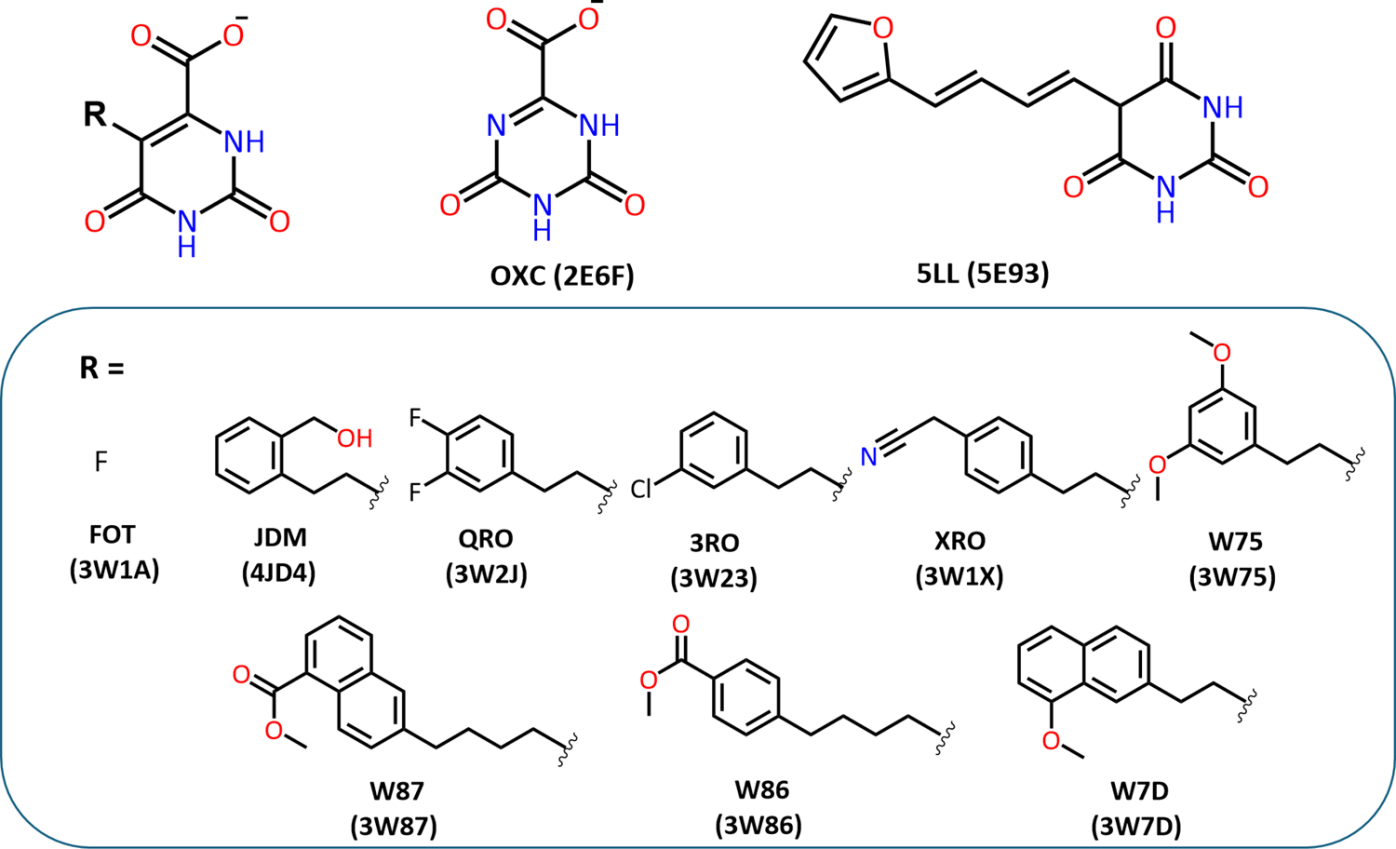
*Tc*DHODH inhibitors’ structures and PDB ID.

We selected 11 ligand-*Tc*DHODH complexes from the Protein Data Bank (PDB) (Figure 1). These complexes included an oxonic acid (OXC), a barbituric acid derivative (5LL), and nine orotate acid derivatives (FOT, JDM, QRO, 3RO, XRO, W75, W86, W87, and W7D).^9,17,20,23^ A common feature among these compounds was their binding to a protein site presenting a highly flexible loop between Leu128 and Ala140. This loop plays a crucial role in regulating the opening and closing of the active site.

The protein’s active site is formed for S1–S5 subsites, with subsites S1–S3 closest to the active cavity.^9,21^ Visual inspection of the structures shows that the hydrogen bonding interactions close to the FMN cofactor are performed mainly with residues from the S1 site (Lys43, Asn67, Met69, Gly70, Leu71, Asn132, Asn194, and Asn195) and S2 site (Asn127) by oxonic acid, barbituric acid, and orotate nuclei. As the substituent linked to the barbituric and orotate nuclei increases, hydrophobic interactions with residues from the S2 (Ser44 and Ser99) and S3 (Ser53, Ser68, Leu 101, and Lys214) sites are observed. In addition, the volume increase of the ligands, related to the linker groups used between the aromatic ring and the barbituric (1,3-pentadiene) and orotate (ethylene or butylene) nuclei, influences the mobility of the loop between Leu128 and Ala140, resulting in three types of conformations for the active site: closed, semi-open, and open (Table 1).^20^

**Table 1:** Crystallographic Structure Data of the 11 Selected Ligand-*Tc*DHODH Enzyme Complexes from PDB

**PDB ID**	**ligand**	**p***K*_**i**_	**ligand volume (Å**^**3**^**)**	**active loop**	**Pro134-Lys214 distance (Å)**	**active site volume (Å**^**3**^**)**
**3C3N**	**HOLO**			closed	7.04	245
**2E6F**	**OXC**	4.02	168.75	closed	7.15	218
**3W1A**	**FOT**	4.71	162.00	closed	7.15	217
**5E93**	**5LL**	6.53	218.70	closed	7.66	273
**4JD4**	**JDM**	5.51	234.90	open	17.19	709
**3W2J**	**QRO**	5.71	210.56	semi-open	13.00	665
**3W23**	**3RO**	5.79	219.52	semi-open	13.13	543
**3W1X**	**XRO**	6.12	211.41	semi-open	12.97	665
**3W86**	**W86**	6.12	278.40	semi-open	13.13	628
**3W87**	**X87**	6.32	296.67	semi-open	13.18	613
**3W75**	**W75**	6.53	227.36	semi-open	12.40	535
**3W7D**	**W7D**	7.34	261.00	semi-open	12.96	696

OXC and FOT, with volumes below 170 Å^3^, exhibit a closed binding site conformation characterized by a Pro134–Lys214 distance of 7.15 Å (Table 1). 5LL, despite its larger volume (218 Å^3^), also adopts a closed conformation.^21^ In contrast, seven orotate derivatives with volumes of 210 to 296 Å^3^ (QRO, 3RO, XRO, W75, W7D, W86, and X87) exhibited a semi-open conformation, with Pro134–Lys214 distances ranging from 12.4 to 13.18 Å, although JDM (234 Å^3^) is reported to display an open conformation of the active site, with a Pro134–Lys214 distance of 17.19 Å.^21^

Ligands that increase the active site volume of the *Tc*DHODH enzyme, leading to semi-open or open conformations, generally exhibit a satisfactory inhibition profile (pKi > 5.0).^8^ This trend is reflected in the active loop distance, as well. It was observed that a closed conformation is defined by an active site volume below 300 Å^3^ and a loop distance below 7.7 Å, while semi-open conformations are associated with volumes ranging from 535 to 696 Å^3^ and approximately 13 Å. Open conformations are characterized by a volume exceeding 700 Å^3^ and a distance exceeding 17 Å.

### Molecular Dynamics Simulations

Using GROMACS software with the Charmm36 force field, we performed 100 ns molecular dynamics simulations in triplicate in aqueous systems to assess conformational changes of *Tc*DHODH induced by different inhibitors.^25^ We analyzed the root-mean-square deviation of Cα atoms (RMSD-Cα) of amino acids, the cofactor (RMSD-cofactor), and the ligands (RMSD-ligand) (Figure S1), calculated as the average of triplicate results (Table 2 and Table S1).

**Table 2:** Average Root Mean Square Deviation (RMSD) Values of the Cα Atoms and the Cofactor from the Holoprotein, Protein–Ligand Complexes, and Individual Ligands

**ligand**	**RMSD-Cα (Å)**	**RMSD-cofactor (Å)**	**RMSD-ligand (Å)**
**HOLO**	1.59 ± 0.12	2.27 ± 0.39	-----
**OXC**	1.81 ± 0.07	1.96 ± 0.15	1.77 ± 0.45
**FOT**	1.82 ± 0.06	2.09 ± 0.12	1.85 ± 0.22
**5LL**	1.79 ± 0.10	1.85 ± 0.13	2.14 ± 0.23
**JDM**	1.79 ± 0.06	2.21 ± 0.15	3.45 ± 0.38
**QRO**	1.72 ± 0.05	2.45 ± 0.09	3.61 ± 0.18
**3RO**	1.79 ± 0.11	1.81 ± 0.09	3.81 ± 0.49
**XRO**	1.70 ± 0.07	2.08 ± 0.16	3.08 ± 0.23
**W75**	1.94 ± 0.07	2.11 ± 0.10	3.11 ± 0.35
**W7D**	1.51 ± 0.05	1.58 ± 0.10	1.57 ± 0.36
**W86**	1.89 ± 0.15	2.24 ± 0.11	2.96 ± 0.75
**W87**	2.02 ± 0.05	2.12 ± 0.12	3.58 ± 0.51

Molecular dynamics simulations (MDS) were performed to assess the stability of *Tc*DHODH in its holo form and complex with inhibitors. The convergence of the protein structure, evaluated by RMSD-Cα (Table 2), stabilized within the initial 20 ns of the simulation, and was therefore excluded from the analysis to ensure equilibration.^25,26^ The holoprotein exhibited a mean RMSD-Cα value (1.59 ± 0.12 Å) lower than that of the inhibitor-bound structures. The mean RMSD-Cα for inhibitor-bound structures ranged from 2.03 ± 0.07 Å (FOT) to 2.17 ± 0.13 Å (3RO).

The RMSD-cofactor values (Table 2) indicate that the cofactor has shifted from its initial position of 1.58 Å (W7D) to 2.45 Å (QRO). However, it remained stable within the active site, as indicated by low standard deviations: less than 0.20 Å in the presence of inhibitors and 0.40 Å for the holoenzyme.

RMSD-ligand analysis (Table 2) revealed differences based on ligand volume. Inhibitors with volumes less than 200 Å^3^ exhibited mean and standard deviation values below 2 and 0.5 Å, respectively. In contrast, larger ligands (volume >200 Å^3^) had higher mean RMSD values, ranging from 2.96 to 3.81 Å, except W7D (1.57 Å). The standard deviation of the orotate-derived ligands with an ethylene spacer (JDM, QRO, 3RO, XRO, W75, and W7D) remained below 0.5 Å, indicating stability within the active site. The ligands W86 and W87, which have a butylene spacer, exhibited greater mobility (standard deviations of 0.75 and 0.51 Å, respectively). Despite this mobility, none of the ligands showed a tendency to exit the active site during the simulation time.

After evaluating the global movement of the holoenzyme and ligands, we conducted root-mean-square fluctuation (RMSF) analysis to assess local fluctuations, as this allows for the observation of residue flexibility throughout the simulation.^26^

**Figure 2:**
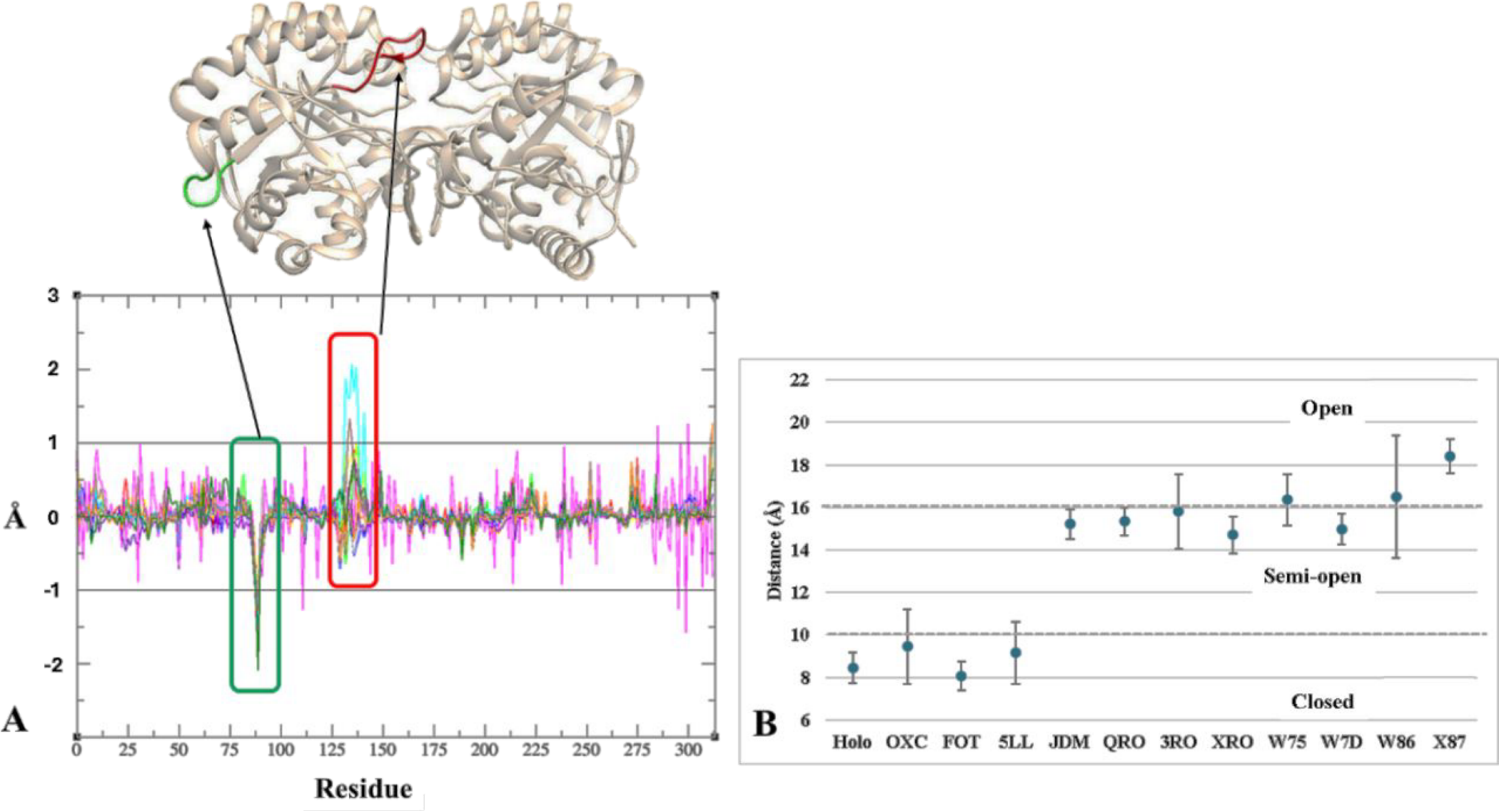
Analysis of structural mobility. (A) ΔRMSF analysis relative to molecular dynamics simulation for all ligands. (B) Distance values for the active loop (average and standard deviation) and the corresponding regions of the active site (closed, semi-open, and open).

We considered the difference between the RMSF of the enzyme’s Cα in the holo form with and without the ligand (ΔRMSF = RMSF ligand – RMSF holo) to evaluate the influence of the ligands within the cavity. Positive and negative values indicate increased and decreased residue mobility, respectively^27,28^ (Figure 2A).

Two regions exhibited ΔRMSF values greater than 1 Å, located between residues His87–Pro93 (green in Figure 2A) and Leu128–Gln138 (red in Figure 2A). While the His87–Pro93 loop, located distantly from both the active site and the dimer interface (>7 Å), showed decreased mobility upon ligand binding, it appears unrelated to the enzymatic action mechanism. Conversely, the active loop region (Leu128–Gln138) displayed variations exceeding 1 Å in W86 and 3RO, compared to ±1 Å variations in other conditions, indicating high and medium residue mobility, respectively. This increased mobility of residues in the active loop likely plays a crucial role in substrate/product entry and/or exit during enzyme activity, potentially leading to a loss of enzymatic activity.^11^

The active loop motion analysis considered the average distance values with a deviation of 3 Å to classify the active site conformations as closed, semi-open, and open (Figure 2B). While the ligands OXC, FOT, and 5LL generally maintain a closed active site conformation, OXC and 5LL exhibited semi-open conformations during the MDS. For JDM, the semi-open conformation appears to be predominant, differing from the open conformation observed in the crystallographic structure (PDB ID: 4JD4). The ligands QRO, XRO, and W7D tend to present a semi-open conformation, while 3RO and W75 exhibit conformations in both semi-open and open states, as demonstrated by the mean standard deviation value of 1 Å. In the case of W86, a mean standard deviation value close to 3 Å suggests high mobility of the active loop, favoring the open conformation. Finally, for W87, the values indicate a predominantly open conformation with a mean standard deviation below 1 Å.

This analysis allows us to observe that ligands promoting a closed conformation of the active site exhibit low biological activity values, whereas ligands that tend to expose the active site more effectively result in better inhibition outcomes. Indeed, evaluating only the volume of the active site is insufficient to predict effective enzyme inhibitors; it is also necessary to determine the intermolecular interactions involved.

The evaluation of hydrogen bond interactions reveals that, in general, oxonic, barbituric, and most orotate derivatives form interactions with residues located in the S1 subsites (Asn67, Met69, Gly70, Leu71, Asn194, and Ser195) and S2 subsites (Asn127 and Ser129), except for the barbituric derivatives 3RO and W86, which interact with just one of these subsites (Table S2).

**Figure 3:**
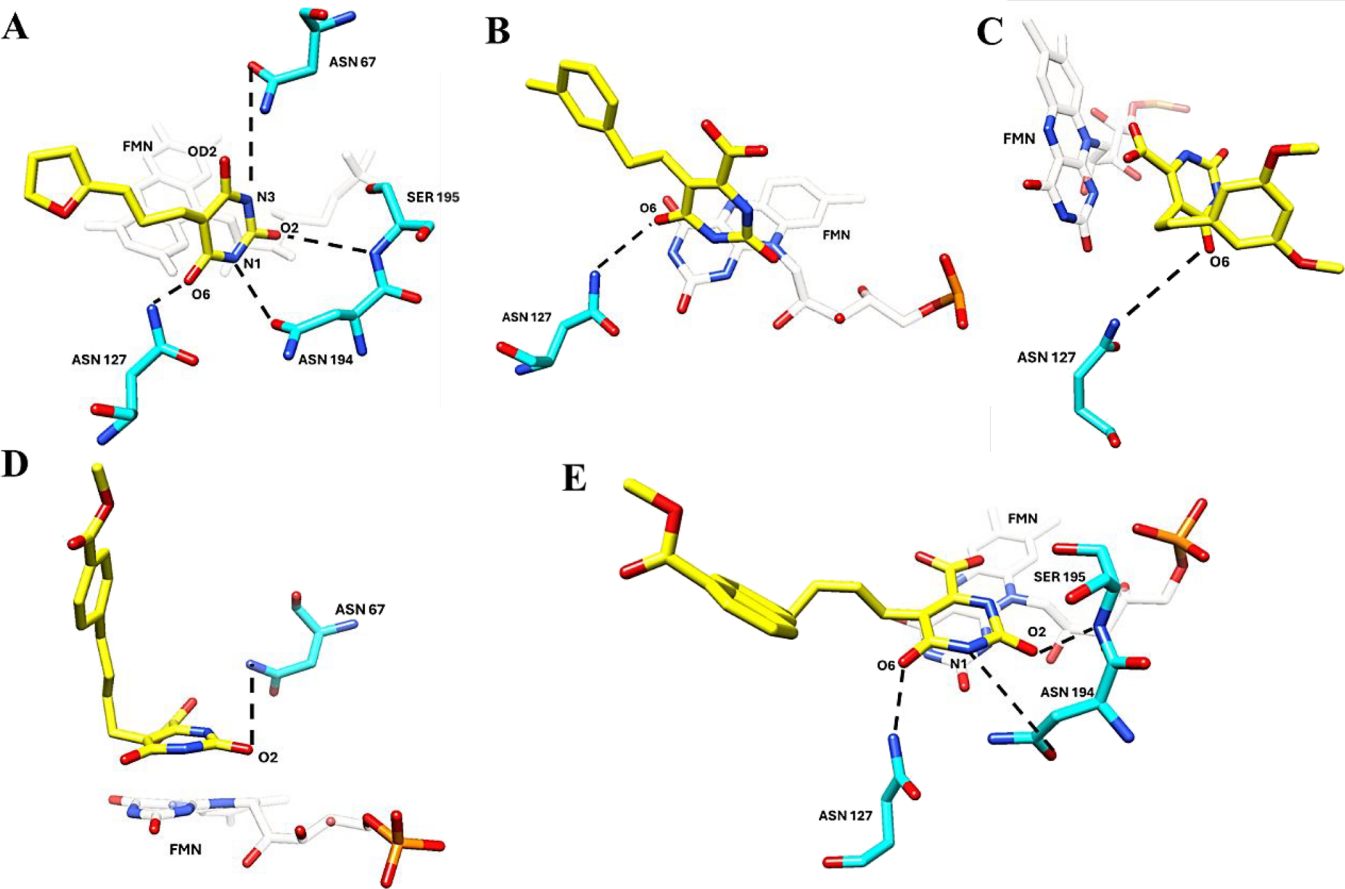
Ligand hydrogen bonding interactions with residues in the *Tc*DHODH active site (A–E). (A) 5LL, (B) 3RO, (C) W75, (D) W86, and (E) W87. The dashed lines represent the hydrogen bonds formed in molecular simulations.

Ligands 5LL (closed), 3RO (semi-open), W75, and W86 (open) exhibit fewer persistent hydrogen bonds and display an average deviation in the distances between Pro134 and Lys214 greater than 1 Å (Figure 3). This result may indicate that the movement of the active loop is influenced by the type of substituent on the inhibitors, affecting the number of hydrogen bonds during the MDS. An opposite effect may occur, where the presence of bulky groups hinders these interactions, as observed with inhibitors W86 and W87, which form fewer hydrogen bonds.

### Binding Energy Calculation

During the molecular dynamics simulation, we calculated the binding free energy (Δ*G*_bind_) for the ligands (derivatives of oxonic, barbituric, and orotate acids). The values of Δ*G*_bind_ and −*T*Δ*S* (entropy) indicate spontaneous interactions between the ligands and enzyme (Table 3).

**Table 3:** Entropy (−*T*Δ*S*) and Binding Free Energy Terms Obtained from the MM-PBSA Calculations Relative to the Binding of Oxonic, Barbituric, and Orotate Derivatives, with the *Tc*DHODH^a^

		**ENERGY**(kcal/mol)				
**#**	-*T*Δ*S*	**Δ*E***_**vΔdW**_	**Δ*E***_**elec**_	**Δ*E***_**solv**_	**Δ*E***_**SASA**_	**Δ*G***_**bind**_
**OXC**	14.00	–19.81 ± 2.65	–21.60 ± 2.47	44.75 ± 2.52	–2.06 ± 0.09	1.27 ± 2.56
**FOT**	56.57	19.50 ± 2.61	–125.14 ± 2.90	93.61 ± 2.46	–2.13 ± 0.10	–14.16 ± 3.07
**5LL**	20.60	–34.29 ± 2.73	–131.06 ± 3.29	115.90 ± 3.06	–3.57 ± 0.10	–53.01 ± 3.22
**JDM**	42.84	–30.88 ± 2.25	–111.63 ± 3.41	83.56 ± 3.26	–3.35 ± 0.14	–62.31 ± 2.96
**QRO**	24.77	–26.95 ± 1.85	–69.98 ± 2.89	50.52 ± 3.88	–3.39 ± 0.13	–49.80 ± 3.16
**3RO**	72.77	–33.00 ± 2.38	–97.98 ± 3.27	58.77 ± 2.97	–3.30 ± 0.15	–75.52 ± 3.30
**XRO**	38.13	–29.77 ± 2.19	–71.91 ± 2.82	53.04 ± 3.51	–3.64 ± 0.17	–52.29 ± 3.54
**W75**	20.64	–31.79 ± 2.32	–115.47 ± 4.15	94.80 ± 4.93	–3.68 ± 0.15	–56.14 ± 3.61
**W7D**	11.53	–27.45 ± 1.81	–66.91 ± 3.32	46.30 ± 3.10	–3.12 ± 0.11	–51.18 ± 2.14
**W86**	30.81	–36.32 ± 2.67	–124.62 ± 3.81	105.25 ± 3.19	–4.28 ± 0.15	–59.98 ± 3.29
**W87**	23.25	–31.63 ± 2.03	–67.10 ± 3.10	50.42 ± 3.90	–3.98 ± 0.18	–52.29 ± 3.08

a Energetic components: van der Waals (Δ*E*_vdW_), electrostatic (Δ*E*_elec_), solvation (Δ*E*_solv_), and solvent-accessible surface area (Δ*E*_SASA_).

Analysis of inhibitor structures maintaining the active site in the closed conformation (Figure 1 and Table 1) revealed distinct interaction profiles. The orotate derivative (FOT), featuring a fluorine substituent, exhibited enhanced electrostatic interactions compared to oxonic acid (OXC) despite a less favorable van der Waals interaction. On the other hand, OXC presented a positive Δ*G*_bind_ value, but the interaction with the active site appears favorable due to entropic factors (−*T*Δ*S* = 14.00). Although the barbituric acid derivative (5LL) presented a lower −*T*Δ*S* value than did FOT, it exhibited enhanced values for electrostatic and van der Waals interactions.

The inhibitor structures maintaining the active site in the open conformation (W75, W86, and W87) presented Δ*G*_bind_ values ranging from −59.98 to −52.29 kcal/mol, while the inhibitors in the semi-open active site conformation (JDM, QRO, 3RO, XRO, and W7D) ranged from −49.80 to −75.52 kcal/mol. These compounds show Δ*E*_vdW_ and Δ*E*_SASA_ values similar to those of the compound 5LL.

JDM (phenylmethanol) and W7D (1-methoxynaphthalene) have substituents with greater and lesser solvent accessibility, respectively. The difference in solvent accessibility likely affects the solvation layer of these compounds, which could influence the distance between residues Pro134 and Lys214 within the active loop. The presence of the butylene spacer in W86 and W87 appears to contribute to an increase in the active site volume without significantly altering the intermolecular interactions.

The van der Waals contributions for W75 (Δ*E*_vdW_ = −31.79 kcal/mol) and W87 (Δ*E*_vdW_ = −31.63 kcal/mol) are similar to those inhibitors in the semi-open active site conformation (Δ*E*_vdW_ from −30.88 to −26.95 kcal/mol), whereas W86 exhibits a more favorable value (Δ*E*_vdW_ = −36.32 kcal/mol). The wide range of electrostatic contribution values (Δ*E*_elec_) appears to correlate with the polarity of the substituents. Those with halogenated phenyl, methyl cyano-phenyl, and naphthyl motifs presented Δ*E*_elec_ values ranging from −66.91 to −97.98 kcal/mol, while the most polar ones presented values below −111 kcal/mol.

The results indicate that although the energetic components provide information about ligand-protein binding, the relationship between Δ*G*_bind_ and pKi appears to be modulated by conformational flexibility and solvent accessibility, emphasizing the need for integrative analyses in rational drug design.

**Figure 4:**
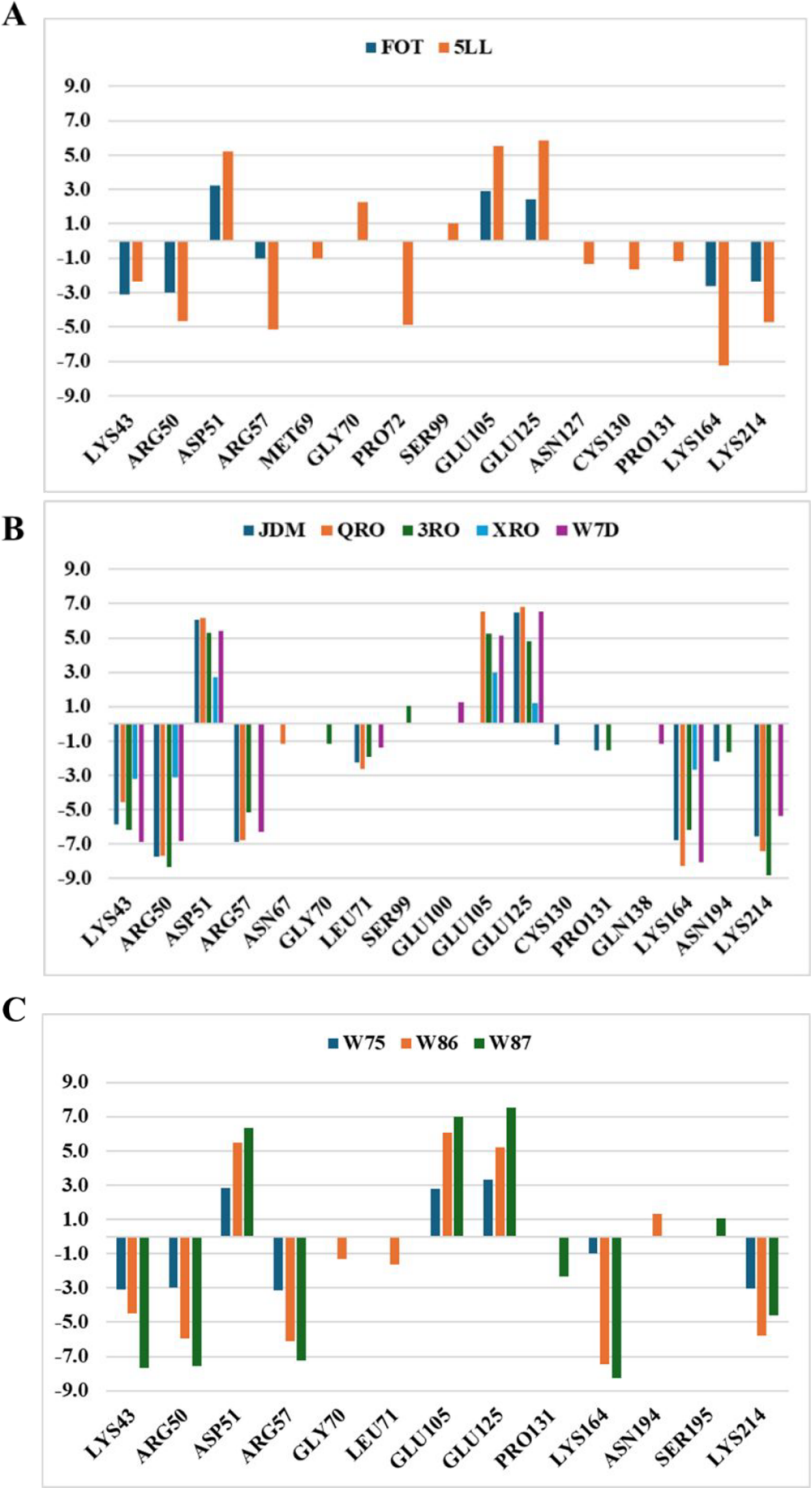
Analysis of binding free energy (Δ*G*_bind_) per residue for the DHODH site in the closed (A- FOT and 5LL), semi-open (B- JDM, QRO, 3RO, XRO, and W7D), and open (C- W75, W86, and W87) conformations.

We assessed the contribution of each residue considering a modular value of 1 kcal/mol (Figure 4). In general, favorable interactions occur with residues located in the S1 (Lys43, Arg50, and Arg57), S2 (Lys164), and S3 subsites (Lys214).^9,21^ The interactions of the closed conformation of the active site were close to those of the open conformation, but their per residue values were lower than those of the closed conformation. The semi-open conformation of the active site exhibited an additional interaction with Leu71 in the S2 subsite and displayed higher interaction values with the S3 subsite (Lys214) compared to the other conformations. The favorable interactions are similar for the most active ligand (W7D) and the least active ligand (OXC). However, the increased interactions with S1 and S3 subsites suggest that enhancing activity.

## Conclusions

This study provides a comprehensive understanding of the structural and dynamic determinants governing the inhibitory activity of the *Tc*DHODH ligands. Our analysis revealed that the volume and structure of ligands significantly influence the active site conformation, with larger ligands inducing more open conformations that correlate with higher inhibitory potency. Molecular dynamics simulations further supported these findings, highlighting the importance of ligand-induced conformational changes and the role of key hydrogen bonds in stabilizing the ligand–enzyme complex. The RMSF analysis identified critical regions involved in flexibility and substrate/product dynamics, emphasizing the dynamic nature of the active site. By elucidating these key factors, this study offers valuable insights into the rational design of *Tc*DHODH inhibitors, paving the way for the development of novel therapeutic strategies targeting this enzyme.

## Methods

### Selection of *Tc*DHODH Structures

The experimental X-ray crystallography data of *Tc*DHODH protein were obtained by searching the Protein Data Bank (PDB) of the Research Collaboratory for Structural Bioinformatics (RCSB).^29^ Using the term ″dihydroorotate dehydrogenase (fumarate)″ by polymer entity description, the organism *T. cruzi* strain CL Brener and structures with a resolution lower than 2 Å. The following crystals and their respective ligands were chosen: 2E6F (OXC),^17^ 3W1A (FOT),^9^ 5E93 (5LL), 4JD4 (JDM), 3W2J (QRO), 3W23 (3RO), 3W1X (XRO), 3W86 (W86), 3W87 (W87), 3W75 (W75), and 3W7D (W7D).^23^

No adjustments were necessary for the crystals for missing residues in all 11 crystallized structures, and water molecules and crystallization residues (*e.g*., glycerol and cobalt hexamine(III)) were removed. The p*K*_i_ value was calculated for the ligands as the negative logarithm of the *K*_i_ value obtained in nanomolar units from the respective articles.^9,17,23^

### Molecular Dynamics Simulation

The methodology involved several steps to prepare and simulate the protein–ligand complex. Initially, crystal protein structures were adjusted to a pH of 7.4, and hydrogen atoms were added using Avogadro software.^30^ Subsequently, the structures of the ligands and cofactors were extracted from the crystals for subsequent procedures. The enzyme structures obtained from the PDB server were submitted to the APBS server (https://server.poissonboltzmann.org)^31^ to predict the p*K*_a_ of the residues. Then, the resulting protonation information was used to add hydrogen atoms to the structures using the Avogadro software.^30^

Ligand and cofactor parameters were obtained from the CGenFF server by loading the mol2 files using the server’s standard configuration. The ligands were inserted into the protein chain A. We acquired the str format from this data, and a script was employed to generate the itp and prm files, which are essential for creating the topology of the ligands and cofactors.^32^ These parameters were then incorporated into the topology and pdb files of the protein, resulting in a final molecular dynamics model employing the CHARMM 36 force field.^33^ The initial steps of molecular dynamics simulations were conducted using GROMACS 2021.^34^

The protein–ligand complexes were inserted into a periodic boundary condition based on a triclinic box filled with TIP3P water molecules, ensuring a minimum distance of 14 Å between the protein and the edge of the box. To neutralize the system charge, nine Na^+^ ions were added. Newton’s equations of motion were integrated using the leapfrog scheme with a time step of 2 fs.^35^ The system then underwent two rounds of minimization, first with positional restrictions and then without using the steep algorithm. Subsequently, equilibration steps were carried out in the NVT and NPT ensembles with a constant temperature (300 K) and pressure (1 bar) followed by a production run of 100 ns. The Lincs algorithm was applied to constrain bond lengths involving hydrogen atoms. The electrostatic measurements were calculated using the long-range particle-mesh Ewald method (PME) with a cutoff distance of 12 Å. The van der Waals interactions were evaluated using the short-distance Verlet algorithm with a switching function between 10 and 12 Å. All simulations were performed in triplicate with a random seed. The first 20 ns of the 100 ns simulation were not considered for analysis and are not represented in the time series.

Simulations were conducted in triplicate, and the values obtained in the following analyses were derived from the averages of the three trajectories. All the molecular dynamics analyses were performed using the GROMACS 2021 software, with the modules gmx_rms, gmx_gyrate, gmx_rmsf, gmx_hbond, gmx_dist, which generate the respective values for RMSD (root-mean-square deviation), radius of gyration (Rg) and RMSF (root-mean-square fluctuation), number of hydrogen bonds, and distance between selected atoms (Pro134Cα–Lys214Cα). The frequency of the hydrogen bonds was calculated using the HbMap2Grace software.^36^

RMSD values were calculated considering the alpha-carbon (Cα) atoms of the protein after square fitting with itself. The cofactor and the ligand were done using the Cα atoms plus cofactor or Cα atoms, cofactor plus ligand, following a square fitting with cofactor or ligand, respectively. The relative difference of RMSF (ΔRMSF) and the hydrogen bond analysis were performed for the last 80 ns of the MDS trajectory. The ΔRMSF was calculated considering the average of DHODH in the holoprotein form against the average of ligand-DHODH. The average H-bonds (D–H···A) were computed by assuming the cutoff distance between donor (D) and acceptor (A) atoms until 0.40 nm and the cutoff H–D–A angle until 30°. The H-bond frequency was calculated using the hbmap2grace package.

To ensure the selection of thermodynamically representative frames for the binding free energy calculations, a conformational cluster analysis was performed using the gmx_cluster module of GROMACS. The GROMOS algorithm^37^ was applied with a cutoff of 0.1 nm for the RMSD of the Cα atoms, starting from 20 ns, excluding the initial equilibrium phase. The most populated cluster representing the structurally predominant conformation was selected as the reference configuration.

The binding free energy of the representative poses of the ligands was also calculated using the MM-PBSA (Molecular Mechanics/Poisson–Boltzmann Surface Area) tool, which calculates different types of energy terms such as van der Waals, electrostatic energy, polarization energy, and solvent-accessible surface area (SASA) between the ligand and protein.^38^

To calculate the entropy (−*T*Δ*S*), we use the interaction entropy method^39^ available in the gmx_MMPBSA tool,^40^ based on MM-PBSA.py, considering the following parameters: PBRadii = 7; interaction_entropy = 1; ie_segment = 50; and temperature = 300.

The XMGRACE software^41^ and the CHIMERA 1.15 software^42^ were used to create the graphs and images, respectively. The visual inspection of the structures was performed using Chimera software, which allowed for the detailed visualization and interpretation of molecular interactions. The volume of the ligands was calculated using the VMD program^43^ and the CASTp 3.0 server,^44^ which specializes in identifying and measuring cavities, pockets, and channels in proteins.

## Supporting Information Available

(Supplemental Table 1) Alpha-carbon distances (Å) between residues Pro134 and Lys214, obtained from molecular dynamics simulation of the enzyme in its holo state and in complex with ligands; (Supplemental Table 2) hydrogen bond donors and acceptors with >50% persistence in the molecular dynamics trajectories (performed in triplicate) of 11 *Tc*DHODH-inhibitor complexes; (Supplemental Figure 1) average RMSD plots for the triplicate MD simulation runs of protein (chain A) along the trajectory (PDF)

## Abbreviations

CYP51: sterol 14-demethylase
DHODH: dihydroorotate dehydrogenase
*Hs*DHODH: *Homo sapiens* dihydroorotate dehydrogenase
*Tc*DHODH: *Trypanossoma cruzi* dihydroorotate dehydrogenase
ΔRMSF: relative difference of RMSF
Δ*E*_vdW_: difference in van der Waals energy
Δ*E*_elec_: difference in electrostatic energy
Δ*E*_solv_: difference in solvation energy
Δ*E*_SASA_: difference in solvent-accessible surface area energy
FMN: flavin mononucleotide
Δ*G*_bind_: difference in binding free energy
MDS: molecular dynamics simulation
MM-PBSA: Molecular Mechanics/Poisson–Boltzmann Surface Area
PDB: Protein Data Bank
PME: particle-mesh Ewald method
RMSD: root-mean-square deviation
RMSF: root-mean-square fluctuation
Rg: radius of gyration
SBDD: structure-based drug design
TPI: triosephosphate isomerase.
