# Supplementary material for: Molecular Dynamics Studies on Trypanosoma cruzi Dihydroorotate Dehydrogenase Complexes: An Analysis of the Inhibitor Influence
Source: ACS Omega. 2025 Apr 25;10(17):18116–24. doi: 10.1021/acsomega.5c01872 (PMC12060061; doi:10.1021/acsomega.5c01872)

Molecular dynamics studies on *Trypanosoma cruzi* dihydroorotate dehydrogenase complexes: An analysis of the inhibitor influence

Eldio G. Santos,[1] Luiz A. P. Flores-Junior,[1] Camilo H. S. Lima,[2,*] Luiza R. S. Dias[1,*]

[1] Laboratório de Química Medicinal, Departamento de Tecnologia Farmacêutica, Faculdade de Farmácia, Universidade Federal Fluminense, 24241-000, Niterói, RJ, Brazil

[2] Laboratório de Modelagem Molecular, Departamento de Química Orgânica, Instituto de Química, Universidade Federal do Rio de Janeiro, 21941-909, Rio de Janeiro, RJ, Brazil

Supplemental Table 1. Alpha-carbon distances (Å) between residues Pro134 and Lys214, obtained from molecular dynamics simulation of the enzyme in its holo state and in complex with ligands.

#	pK_i	DISTANCE (Å)	CONFORMATION
**HOLO**	-	8.46 ± 0.71	Closed
OXC	4.02	9.45 ± 1.76	Closed
**FOT**	4.71	8.0 7± 0.67	Closed
**5LL**	6.53	9.15 ± 1.45	Closed
**JDM**	5.51	15.21 ± 0.68	Semi-open
**QRO**	5.71	15.35 ± 0.66	Semi-open
**3RO**	5.79	15.81 ± 1.76	Semi-open
**XRO**	6.12	14.70 ± 0.88	Semi-open
**W75**	6.53	16.37 ± 1.21	Open
**W7D**	7.34	14.98 ± 0.71	Semi-open
**W86**	6.12	16.51 ± 2.88	Open
**W87**	6.32	18.41 ± 0.81	Open

Supplemental Table 2. Hydrogen bond donors and acceptors with >50% persistence in the molecular dynamics trajectories (performed in triplicate) of 11 *Tc*DHODH-inhibitor complexes.

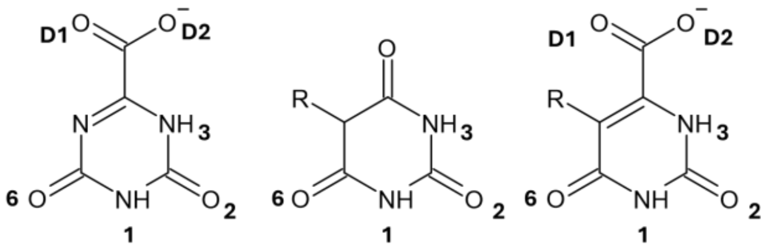

Oxonic **Barbituric** **Orotate**

**#**	**HYDROGEN BOND DONOR**	**ACCEPTOR**	**PERSISTENCE (%)**
**OXC**	Asn67ND2	O2	51.24
	Met69N	OD2	59.19
	Gly70N	OD2; OD1	98.11; 86.98
	Leu71N	OD1	91.06
	Asn127ND2	O6	56.81
	Asn194ND2	O6; N1	63.10; 51.17
	N1	Asn194OD1	52.96
	N3	Asn67OD1	50.71
**FOT**	Met69N	OD1	61.60
	Gly70N	OD2; OD1	84.71; 93.29
	Leu71N	OD2	93.30
	Asn127ND2	O6	59.44
	Asn194ND2	N1	60.04
	N1	Asn194OD1	56.20
**5LL**	Asn67ND2	N3	50.56
	Asn127ND2	O6	50.25
	Asn194ND2	N1	67.73
	Ser195N	O2	58.69
**JDM**	Gly70N	OD2	88.74
	Leu71N	OD2	54.69
	Asn127ND2	O6	57.11
	N1	Ser129O	56.81
**QRO**	Asn127ND2	O6	82.78
	N1	Ser129O	65.12
**3RO**	Asn127ND2	O6	65.15
**W75**	Asn127ND2	O6	56.50
**W7D**	Asn67ND2	O2	87.22
	Met69N	OD2	97.26
	Gly70N	OD2; OD1	100; 97.76
	Leu71N	OD1	100
	Asn127ND2	O6	93.69
	Asn194ND2	N1	87.22
	N3	Asn67OD1	99.20
	N1	Asn194OD1	97.89
**XRO**	Asn127ND2	O6	74.66
	Ser129OG	N1	51.97
	Ser195N	O2	62.57
**W86**	Asn67ND2	O2	53.69
**W87**	Asn127N	O6	82.43
	Ser195N	O2	83.33
	N1	Asn194OD1	57.88

Supplemental Figure 1. Average RMSD plots for the triplicate MD simulation runs of protein (chain A) along the trajectory. (A) the closed state (2E6F - black, 3W1A - Red and 5E93 - green); (B) the semi-open state (4DJ4 - black, 3W2J - red, 3W23 - green, 3W1X - blue and 3W7D - yellow); (C) the open state (3W75 - black, 3W86 - red and 3W87 - green).

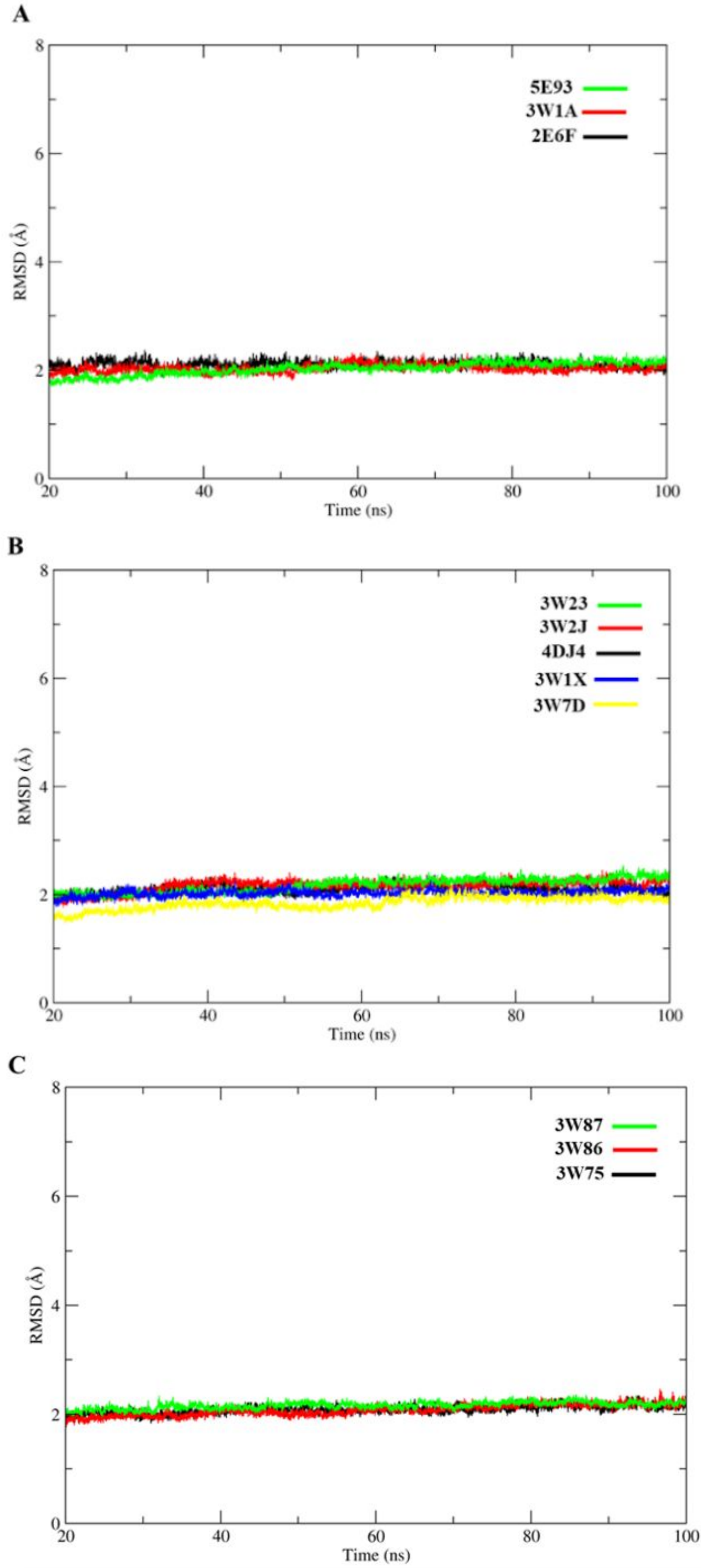

Supplement: Supplementary file 1 — ao5c01872_si_001.pdf [file ao5c01872_si_001.pdf]
